# The Preference and Actual Use of Different Types of Rural Recreation Areas by Urban Dwellers—The Hamburg Case Study

**DOI:** 10.1371/journal.pone.0108638

**Published:** 2014-10-14

**Authors:** Thiemen Boll, Christina von Haaren, Eick von Ruschkowski

**Affiliations:** 1 Leibniz Universität Hannover, Institute of Environmental Planning, Hannover, Germany; 2 Nature and Biodiversity Conservation Union Germany (NABU), Department Nature Conservation and Environmental Policy, Berlin, Germany; University of Hawaii at Manoa, United States of America

## Abstract

In the wake of urbanisation processes and the constitution of metropolitan regions, the role of the city's rural surroundings is receiving more attention from researchers and planners as rural areas offer various (cultural) ecosystem services for the urban population. Urban dwellers increasingly desire recreation and landscape experience. Although this need for recreation is generally recognized, few studies have focused on the question of people's preferences for certain types and characteristics of outdoor recreation areas in relation to the frequency of use. In order to acquire baseline data on this subject, the main objectives of this study were to explore recreation preferences of urban dwellers and the relation between actual use and perceived value of recreation areas in a case study in the Hamburg Metropolitan Region (Germany). In a social survey, Hamburg residents (n = 400) were asked about their preferences and use of four important regional recreation areas with different landscape characteristics in face-to-face interviews in different locations in the city. We found that both outdoor recreation within and outside of the city were fairly or very important for more than 70% of the questioned urban dwellers. Interestingly, the preference for a recreation area outside of the city did not depend on the frequency of use, which indicates that certain recreation areas had a symbolic value besides their use value. When people were questioned on the characteristics of recreation areas, perceived naturalness was found to be strongly related to preference. Respondents considered the diversity, uniqueness, and naturalness of the landscape to be far more important than the accessibility of the recreation areas and the provision of service facilities.

## Introduction

Rural and natural areas in or near metropolitan regions fulfil various functions for the urban population and offer ecosystem services. They provide drinking water, support the regional supply of food as well as renewable energies from wind, water, solar and biomass, regulate and improve the regional climate, mitigate flood risks, contribute to regional identity, and function as recreational area for the urban population [1], [2], [3], [4]. The provision and availability of cultural ecosystem services like recreation and landscape experience can be considered an important location factor in the context of globalisation and cities' competition for new inhabitants, a skilled work force, and tourists [5]. Both parks and green spaces within city limits and the availability of nearby open spaces or landscapes contribute to a healthy living environment for the urban population [6], [7], [8]. There are many positive effects of urban green spaces on human well-being. Living in a greener area for example has a significant effect on mental distress and life satisfaction [9], [10]. People who live in urban areas with more green space tend to report superior well-being than city dwellers without parks, gardens or other green spaces nearby [9].

In many cases, urban green spaces cannot completely fulfil the recreational needs of urban dwellers [11]. Surrounding rural areas with high aesthetic qualities are important recreational areas not only for day trips, but also for weekend recreation. As rural areas often lag behind urban areas economically, infrastructure may be missing. Establishing a recreational infrastructure with specific services for urban recreationists (e.g. restaurants) may provide added value to these areas. Both landscape-related characteristics (landscape aesthetic qualities) and infrastructure and service-related characteristics are relevant for recreational services. Characteristics that are important to describe landscape aesthetic qualities are diversity, uniqueness and naturalness. These characteristics are used in the German Federal Nature Conservation Act (BNatSchG) to describe the value of landscape aesthetics. They are also used for inventorying and assessing landscapes in landscape planning as legally mandated for in the same law [12], [13]. Besides the landscape's aesthetic qualities, place attachment is an important characteristic to identify the personal relationship to the recreation area [14], [15]. Service-related characteristics of recreation areas comprise accessibility, food services, and information services. Hence, understanding the recreational preferences of urban dwellers is of importance to rural municipalities and also to regional and environmental planners as infrastructure and service development could be tailored to the expectations of the users and increase urban-rural interactions in metropolitan regions [16], [17]. More importantly, on the one hand, understanding how and why recreational areas are used could help to develop recreational qualities and infrastructures. On the other, understanding the intrinsic value of a landscape and the bond between the urban population and rural landscapes could serve as a basis for developing urban-rural landscape policies, including land stewardship schemes or activating engagement of urban citizens when competing land uses endanger rural recreational quality.

While the recreational use of areas such as national parks or other protected landscapes has been more or less under constant observation – mainly to mitigate the negative impact of recreational uses on natural resources [18], [19] – rural landscapes for daily or weekend recreation do not receive the same amount of attention, meaning that there is a lack of reliable data. Although general surveys on recreational activities are carried out in some countries [20], they do not provide relevant data for planners, government agencies, and other institutions. This lack of data becomes even more obvious when site-specific or activity-related knowledge is concerned. Existing local planning concepts mostly focus on facility-related recreation and sports [3] and studies concentrate on urban parks [21], [11]. Overall, comparative knowledge concerning the perceived importance and actual use of recreation areas by urban dwellers is very limited.

In order to address the lack of relevant knowledge, the main objectives of the study were to explore recreation preferences of urban dwellers and the relation between actual use and perceived value of recreation areas. The following research questions were examined in detail in a case study in the Hamburg Metropolitan Region:

– How important is outdoor recreation for urban dwellers and where do they carry out recreational activity?
– What is the relation between preference and actual use of recreation areas?
– Which are the most important characteristics of recreation areas?
– How do people evaluate the importance of landscape-related characteristics compared to infrastructure and service-related characteristics?
– How do socio-demographic factors influence outdoor recreation behaviour?

The southern part of the Hamburg Metropolitan Region was chosen as case study because it represents a very diverse urban-rural context. While Hamburg is a big, economically vibrant and dense city with 1.8 million inhabitants, which creates a high recreational demand on the surrounding areas, the surroundings are very rural and include many different landscape types. Though the city of Hamburg itself has the highest regional gross domestic product (GDP) in Germany and the fifth highest in Europe, the NUTS2 region Lüneburg directly adjacent to Hamburg's southern borders is below European average. This strong economic imbalance between urban and rural areas in the region makes the rural areas economically dependent on Hamburg. An important aspect of improving economic performance in the rural areas is to attract more tourists and recreationists [22].

**Figure 1:**
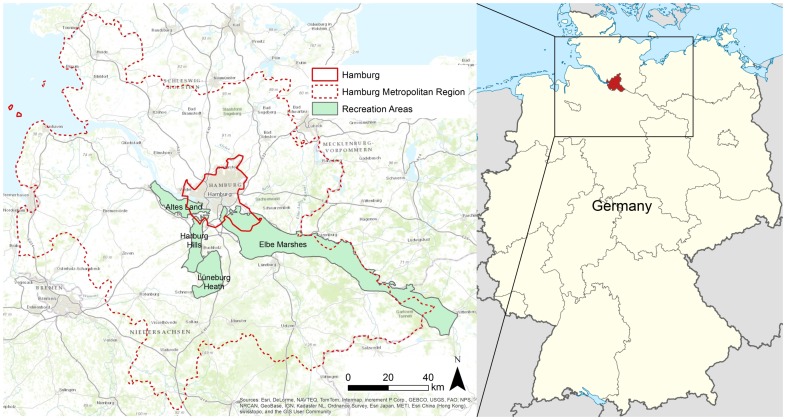
Location of the recreation areas in the Hamburg Metropolitan Region. The four tested recreation areas were the Lüneburg Heath, the Harburg Hills, the Elbe Marshes and the Altes Land.

Due to the metropolitan region's size, the study focuses on its southern part, in which four major recreation areas adjacent to the city were identified by expert discussion: Lüneburg Heath, Harburg Hills, Elbe Marshes and Altes Land (Fig. 1). The natural characteristics of the landscapes and forms of land use are very different among the four recreation areas. Therefore, all recreation areas have a unique landscape character (Table 1). While the Lüneburg Heath, the Altes Land and in parts the Elbe Marshes are well-known historical cultural landscapes [23], the Harburg Hills are not well-known as cultural landscapes. The North Sea coast, certainly a major day-trip recreational destination, was excluded from the study. Instead, we concentrated on regions where the potential of human-introduced landscape changes to support recreation and tourism is much higher. At the North Sea coast, which is a national park and a world heritage site, only few human-induced changes are permitted.

**Table 1:** Characteristics of the Lüneburg Heath, Harburg Hills, Elbe Marshes and Altes Land recreation areas.

Recreation area	Landscape type^1^ and individual description	Relevance for tourism and recreation	Cumulative share of protected areas^2^	Distance from Hamburg
**Lüneburg Heath**	Forest landscape rich in heathland and nutrient-poor grassland; dynamic relief for northern Germany due to glacial processes in the ice ages (up to 169 m); poor, dry and sandy soils, few arable lands, mostly heathland and pine forests	Important tourist destination in northern Germany, heathland of international importance, oldest and largest nature reserve in Lower Saxony	78.22%	Longest distance from Hamburg, approx. 1 h by car
**Harburg Hills**	Forest landscape; dynamic relief for northern Germany due to glacial processes in the ice ages (up to 150 m); few waterbodies, mostly coniferous forest, few natural forests, few protected areas	First range of hills and forest area south of Hamburg, popular local recreation area (e.g. walking, riding, mountain biking)	7.55%	Adjacent to the southern districts of Hamburg
**Elbe Marshes**	Open cultural landscape rich in meadows; floodplain of the Elbe river; many waterbodies, widespread agriculture (mostly meadows, in parts extensive), few forests	Partly belongs to the ‘Elbe Valley’ biosphere reserve, important tourist destination (e.g. cycling)	43.5%	Adjacent to the south-eastern districts of Hamburg, partly within Hamburg
**Altes Land**	Orchard-dominated landscape; flat land adjacent to the Elbe river, protected by dykes; intensive agriculture on fruit plantations and meadows	Largest continuous fruit cultivation area of Central Europe, well-known and popular cultural landscape throughout Germany	12.17%	Adjacent to the south-western districts of Hamburg, partly within Hamburg

1 Classification of landscape types by the German Federal Agency for Nature Conservation (BfN).

2 Protected areas include biosphere reserves, special areas of conservation, special protection areas, nature reserves and landscape protection areas (2010).

## Research Design and Methods

### Ethics statement

The survey was carried out in accordance with legal requirements and was reviewed and approved by the Institute of Environmental Planning and its executive director. At the time of the survey there was no ethics committee at the Leibniz University of Hannover and no further approval was needed to conduct the survey. The survey was voluntary, anonymous and did not include controversial questions. At the beginning of the survey all respondents were informed that the survey was anonymous and the data would only be used for research purposes including publications. All respondents were asked if they agreed and if they wanted to participate in the survey. Oral consent of participants was documented on the questionnaire. People who did not give oral consent were not interviewed.

### Questionnaire

The questionnaire consisted of three parts with closed questions (Appendix S1). Part one focused on general recreational behaviour and frequency of use. Respondents had to specify on a five-point scale whether their outdoor recreation takes place within or outside of the city (Question 1a) and how important each of these areas was to them (Question 1b). The second part dealt with the four study recreational areas: the Lüneburg Heath, the Harburg Hills, the Elbe Marshes, and the Altes Land. For each area, the same set of questions about area knowledge and frequency of use was asked (Questions 2a–d). Additionally, participants had to identify their favourite area of those four. The frequency of use was measured on a scale from ‘never’ to ‘weekly’; frequent users were later defined as those who visited the area(s) at least on a monthly or a weekly basis. The correlation between the variables ‘area knowledge’, ‘recreation area visited at least once’, ‘preferred recreation area’, and ‘frequency of use’ were analysed for all four recreation areas. The third part of the survey addressed the characteristics of respondents' favourite recreation areas (Questions 3a–b). The subsamples thus varied according to the preference for the areas (n = 138 for the Lüneburg Heath, n = 137 for the Altes Land, n = 75 for the Elbe Marshes, n = 50 for the Harburg Hills). The limitation of one recreation area per respondent was chosen because pre-tests revealed that many people did not know all four recreation areas.

Respondents had to assess the recreation areas based on the criteria diversity, uniqueness, naturalness, place attachment, accessibility, food services, and information services. The criteria are subject to the personal situation and the preferences of the participants. While the individual perception of the landscape influences the assessment of diversity, uniqueness and naturalness, accessibility relies on factors such as the availability of a car or public transit. Each criterion was assessed on a 5-point Likert scale (from very low to very high). Questions that respondents could not answer were defined as missing values and not included in the respective analysis. Finally, the socio-demographic variables age, gender, education and place of residence were collected from the participants.

### Survey

The study was designed as a quantitative face-to-face survey and was conducted by five researchers and students of the Leibniz University of Hannover. Survey participants were initially selected on a random sample basis at 12 different locations on 6 different survey days in Hamburg. Interviews were conducted during the week and at the weekend at times from 8 am to 8 pm. Later, in a supplementary round of interviews, the sample was stratified in order to increase participation of underrepresented population groups in terms of age and gender to achieve improved representativeness for Hamburg's population [24]. The desired sample size was set to n = 400 to generate reliable results. The interviews lasted about 10 minutes. Respondents were shown answer cards after each question in order to improve understanding and rating. The answer cards included the possible answers to the closed questions with a five point Likert scale to illustrate equal distances among rating categories. As knowledge about the rural recreation areas around Hamburg was a prerequisite to assess their characteristics, the questionnaire contained a filter question to sort out non-residents, e.g. visitors to Hamburg. We addressed more than 1000 people in order to achieve a sample of 400. The main reasons for not taking part in the survey were lack of time and disinterest.

The term ‘outdoor recreation’ needed to be clarified to respondents to ensure a common understanding. It was explained as ‘recreational activities that take place outdoors, like taking a walk, running, horseback riding, etc., where the attractiveness of nature and landscape plays an important role’ at the beginning of the survey. The survey distinguished between outdoor recreation within Hamburg and outside of Hamburg. Recreation areas within Hamburg include parks and public green spaces like the Alster Lake and Planten & Blomen Park. Outside of Hamburg we focused on the four above mentioned recreation areas.

### Statistical analysis

The IBM SPSS Statistics 19 software was used for data entry and analysis. Answers on a five-point rating scale were analysed with methods for interval-scaled data [25]. Correlations between variables were analysed using Pearson's correlation coefficient (r_P_). Assessment of criteria among recreation areas were analysed using one-way ANOVA (F). Pearson's chi-squared test (χ^2^) was used to test the representativeness of the sample for Hamburg's total population.

## Results

### Sample characteristics

The sample was representative for Hamburg's population in terms of the socio-demographic variables age and gender, while it was not representative for education and residential district [26]. The gender balance was 51.7% female and 48.3% male and did not significantly deviate from the population of Hamburg (χ^2^ = 0.057; df = 1; p = 0.812). The average age of the sample was 44 years (min = 15; max = 85; SD = 17.21), while the average age of the Hamburg population is 42.2 years [26]. The sample did not significantly deviate from the age distribution among age classes (<30 years/ 30–49 years/ 50–65 years/ ≥65 years) of the Hamburg population (χ^2^ = 7.612; df = 3; p = 0.055). The educational level of the sample was significantly higher than that of the Hamburg population (χ^2^ = 81,229; df = 1; p<0.001). While the percentage of people with a general qualification for university entrance was 67% in the sample, it is 44.6% in the population of Hamburg. The distribution of respondents among Hamburg residential districts was not representative for the population of Hamburg (χ^2^ = 97.678; df = 6; p<0.001). The subsamples of respondents who preferred different recreation areas differed significantly in terms of the age classes (χ^2^ = 18.134; df = 9; p = 0.034), while other characteristics were not significantly different. Respondents who preferred the Elbe Marshes were for example significantly older (49.6 years) than respondents who preferred the Harburg Hills (41.8 years).

### Relevance of outdoor recreation

**Figure 2:**
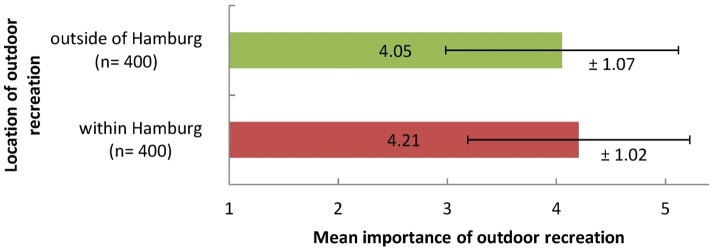
Mean Importance and standard deviation of outdoor recreation within and outside of Hamburg. Respondents assessed on a scale from 1 (not important) to 5 (very important).

Outdoor recreation both within and outside of Hamburg's city limits were very important for the residents. While only few respondents regarded outdoor recreation as not important or slightly important (7.8% within Hamburg; 10.5% outside of Hamburg), most respondents thought that outdoor recreation was very important or fairly important (79.4% within Hamburg; 72.8% outside of Hamburg). On the five-point rating scale (1 =  not important, 2 =  slightly important, 3 =  moderately important, 4 =  fairly important, 5 =  very important) mean importance of outdoor recreation within Hamburg was assessed with 4.21 (SD = 1.02) and outdoor recreation outside of Hamburg with 4.05 (SD = 1.07; Fig. 2). Outdoor recreation within Hamburg had higher importance for Hamburg residents than outdoor recreation outside of Hamburg (Wilcoxon signed-rank test p = 0.030).

The answer combination given by most respondents was ‘very important’ for both recreation within and outside of Hamburg (25.8% of all respondents). Interestingly, there was no significant correlation between the assessment of outdoor recreation within and outside of Hamburg (r_P_ = −0.260; p = 0.603). For example, people who evaluated outdoor recreation within Hamburg as very important often did not evaluate outdoor recreation outside of Hamburg as similarly important (49.8% of these respondents). The same applies to people who evaluated outdoor recreation outside of Hamburg as very important. They often did not evaluate outdoor recreation within Hamburg as similarly important (42.8% of these respondents).

**Figure 3:**
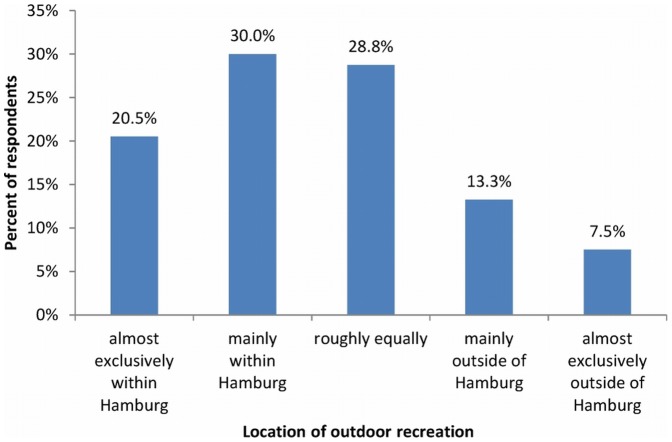
Where outdoor recreation of Hamburg residents takes place (n = 400).

While both the importance of outdoor recreation within and outside of Hamburg were assessed between fairly and very important on average, the actual use was strongly biased towards recreation within the city (χ^2^ = 49.688; df = 1; p<0.001). 50.5% of respondents recreated almost exclusively or mainly within the city, while 20.8% almost exclusively or mainly recreated outside of the city (Fig. 3).

### Knowledge, use and preference of recreation areas

The four recreation areas differed in the share of people who know and who have visited them. The Lüneburg Heath was the best known recreation area (97.5%) and at the same time the recreation area that most people had at least visited once (86.8%). The Altes Land had the second highest level of recognition (90.8%) and second highest level of visitation (83%), followed by the Harburg Hills (known: 85.3%; visited: 73.5%) and the Elbe Marshes which was the least known (76%) and least visited (59.3%) recreation area.

The preference for the four recreation areas in the southern Hamburg Metropolitan Region also differed significantly (χ^2^ = 59.380; df = 3; p<0.001). Having the choice among the four areas, 34.5% preferred the Lüneburg Heath, followed by 34.3% of respondents who preferred the Altes Land. Less favoured were the Elbe Marshes (18.8%) and the Harburg Hills (12.5%).

**Figure 4:**
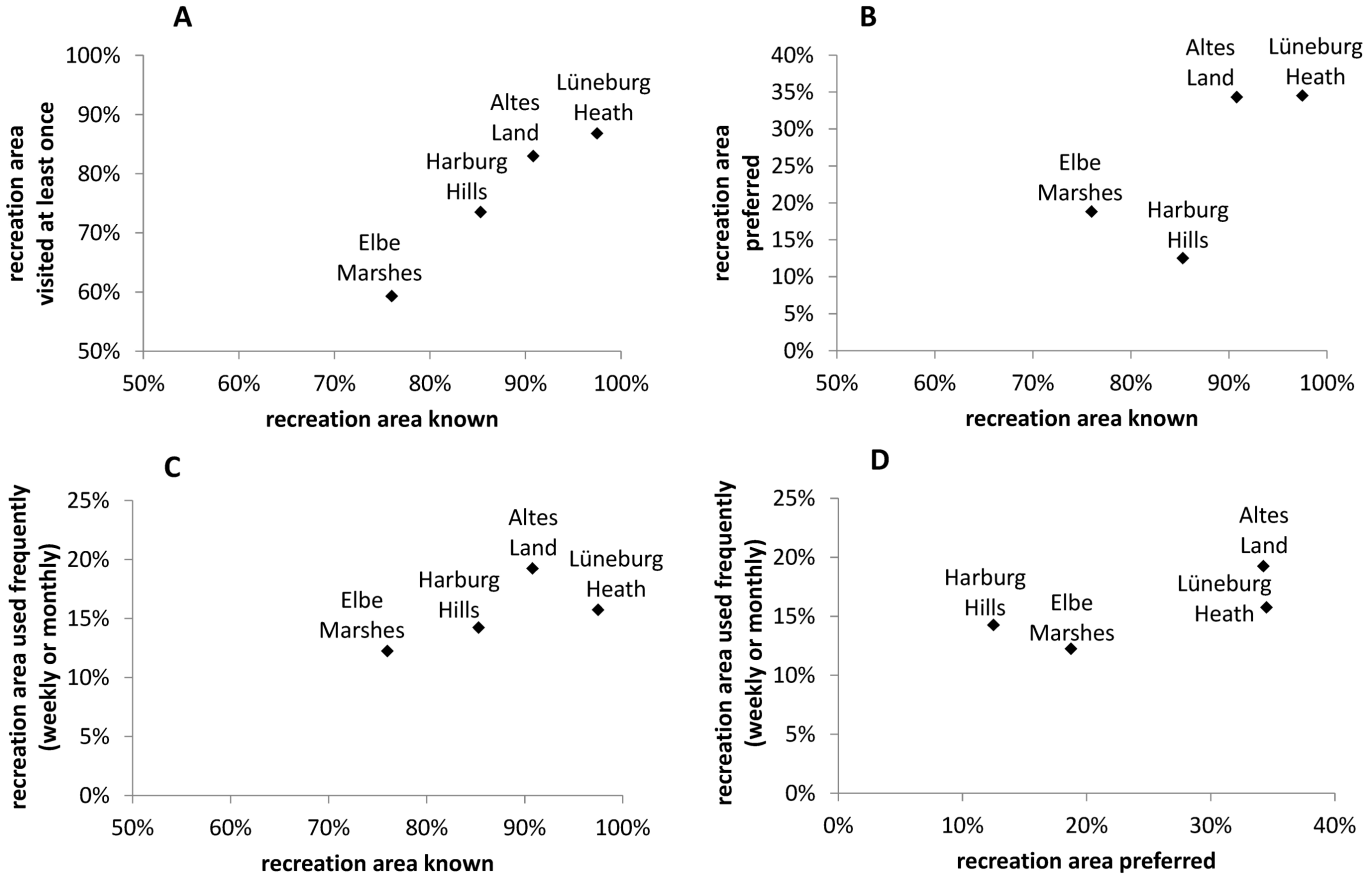
Correlations between area knowledge, preference, visits and frequent use of recreation areas (n = 400). A. Significant correlation between the share of people who know the recreation areas and the share of people who have visited the recreation areas at least once (r = 0.982; p = 0.009 one sided). B. No significant correlation between the share of people who know the recreation areas and the share of people who prefer the recreation areas (r = 0.742; p = 0.129 one sided). C. No significant correlation between the share of people who know the recreation areas and the share of people who use the recreation areas frequently (r = 0.680; p = 0.160 one sided). D. No significant correlation between the share of people who prefer the recreation areas and the share of people who use the recreation areas frequently (r = 0.740; p = 0.130 one sided).

There was strong correlation between the share of people who knew and the share of people who had visited the recreation areas at least once (r_P_ = 0.98; p<0.001; Fig. 4a). The more known a recreation area was, the higher the number of people who had visited it was. Interestingly, the share of people who knew (and who had visited) the recreation areas did not correlate with the preference for recreation areas (r_P_ = 0.74; p = 0.130; Fig. 4b). The Elbe Marshes and the Altes Land were more preferred than expected by the share of people who knew them, while the Lüneburg Heath and the Harburg Hills were less preferred than expected. The non-existing correlation shows that better-known recreation areas are not per se more popular.

Initially, it was assumed that better-known recreation areas would be used more frequently. However, there was no correlation between the share of people who knew a recreation area and the share of people who used it frequently (r_P_ = 0.680; p = 0.160; one sided; fig. 4c). Also, we assumed that more preferred recreation areas were used more frequently. However, there was no significant correlation between preference for an area and frequent use (r_P_ = 0.740; p = 0.130; one sided; Fig. 4d). The share of people who use the Lüneburg Heath frequently was much lower than it was expected by peoples' preference for the area, while the share of people who use the Harburg Hills frequently was much higher than the preference would suggest.

### Most important criteria for recreation areas

**Figure 5:**
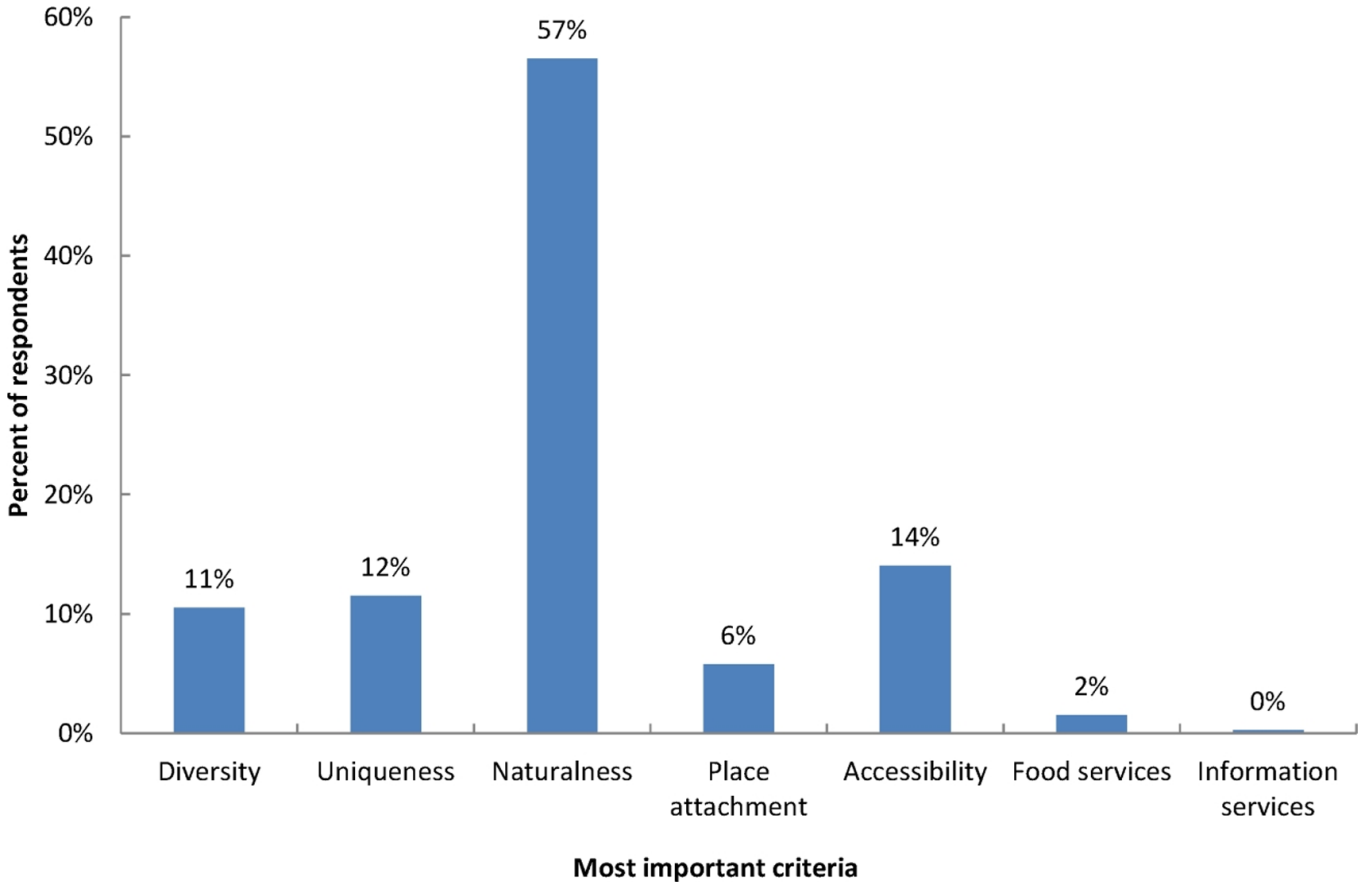
Most important criteria for recreation areas. Respondents were asked to name their most important criterion for recreation areas from the given criteria diversity, uniqueness, naturalness, place attachment, accessibility, food services, and information services.

Respondents regarded naturalness as the most important criterion for recreation areas by far (57%; Fig. 5). Accordingly, landscape-related criteria (diversity, uniqueness and naturalness) were the most important group of criteria (80%) compared to infrastructure and service-related criteria (16%).

There was no significant difference between the preference for a criterion and the preference for a recreation area (χ^2^ = 9.769; df = 15; p = 0.834). For all recreation areas naturalness is by far the most important criterion (Lüneburg Heath 59.4%; Harburg Hills 46.0%; Elbe Marshes 61.3%; Altes Land 54.7%). Also, when analysing each criterion individually, there were no significant differences among recreation areas. This means that the preference for a criterion did not influence the preference for a recreation area or vice versa. However, there were some differences worth mentioning although they were not statistically significant. People who preferred the Harburg Hills (n = 50), an area which is not considered a landscape of extraordinary quality in Germany, did not regard naturalness as such important (46%), whereas accessibility (18%) and diversity (16%) were more important than for people who preferred one of the other recreation areas. People who preferred the Altes Land (n = 75), which can be considered extraordinary because of the vast orchards, put a higher priority on uniqueness (14%), while diversity (7%) achieved the lowest importance in comparison to the other recreation areas.

**Figure 6:**
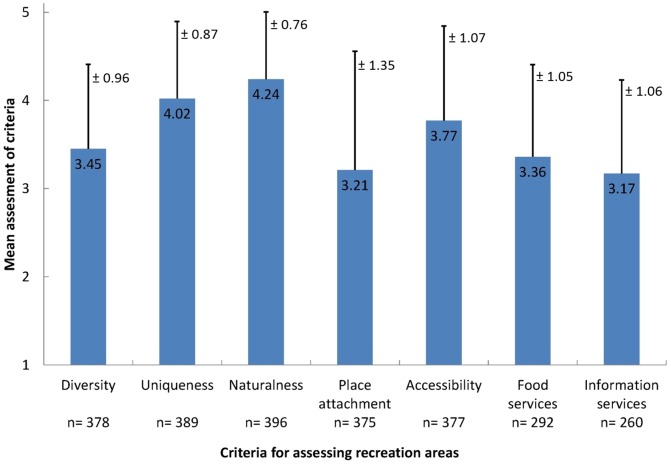
Mean assessment and standard deviation of criteria across all preferred recreation areas. Respondents assessed on a scale from 1 (very low) to 5 (very high).

Naturalness, which was the most important criterion in general, achieved the highest score on the 5-point rating scale across all recreation areas (M = 4.24; Fig. 6). At the same time naturalness had the lowest standard deviation (SD = 0.76) compared to the other criteria. Uniqueness was the second most positively assessed criterion (M = 4.02; SD = 0.87). This means that preferred recreation areas were characterised by positive ratings of uniqueness and naturalness. Respondents were also satisfied with the accessibility of recreation areas (M = 3.77); however, assessments differed more obviously (SD = 1.07). Diversity, contrarily to the other visual landscape quality indicators uniqueness and naturalness, was assessed only moderately (M = 3.45; SD = 0.96). The service indicators ‘food’ and ‘information’ were also assessed moderately (M = 3.36; SD = 1.05 and M = 3.17; SD = 1.06), as was place attachment (M = 3.21). However, the latter was assessed the most different among respondents (SD = 1.35).

The assessments of the landscape-related criteria diversity, uniqueness and naturalness were more consistent among respondents (SD = 0.96; SD = 0.87; SD = 0.76) than the assessments of the criteria accessibility, place attachment, food and information services (SD = 1.07; SD = 1.35; SD = 1.05; SD = 1.06). Additionally, more respondents were able to assess landscape-related criteria (from n = 378 to n = 396) while fewer respondents were able to assess the service-related criteria food (n = 292) and information (n = 260).

### Assessment of criteria among recreation areas

**Figure 7:**
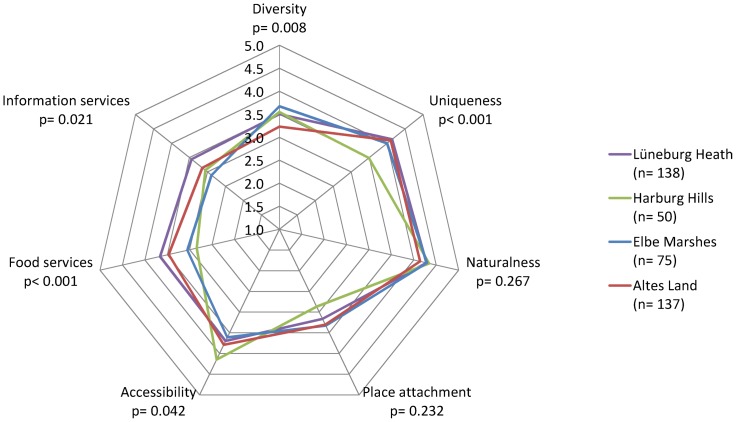
Assessment of criteria among recreation areas. Difference among recreation areas was tested using one-way ANOVA.

Most of the criteria were assessed significantly different among the individual recreation areas (diversity, uniqueness, accessibility, food services and information services), while others were assessed similarly (naturalness and place attachment; Fig. 7). Diversity was assessed significantly different among the recreation areas (df = 3; F = 4.03; p = 0.008). While the Elbe Marshes were assessed as very diverse (M = 3.68; SD = 0.89), the Lüneburg Heath and the Harburg Hills were assessed more moderately (M = 3.50; SD = 0.92 and M = 3.55; SD = 0.87). The Altes Land with its vast orchards achieved the lowest value for diversity (M = 3.23; SD = 1.03).

Uniqueness was assessed significantly different among the recreation areas (df = 3; F = 7.52; p<0.001). In the Harburg Hills, which was the least preferred recreation area, uniqueness was assessed much lower (M = 3.49; SD = 0.96) than in the other recreation areas Lüneburg Heath (M = 4.14; SD = 0.82), Altes Land (M = 4.10; SD = 0.79) and Elbe Marshes (M = 4.00; SD = 0.94). Naturalness was not evaluated significantly different among the recreation areas (df = 3; F = 1.32; p = 0.267). All preferred recreation areas were rated as very natural (from M = 4.14 to M = 4.34; SD from 0.63 to 0.83). This means that high and consistent ratings of naturalness were strongly related to preference.

Accessibility was assessed significantly different among the recreation areas (df = 3; F = 2.76; p = 0.042). While the Harburg Hills, which are located at the southern border of Hamburg and accessible by public transport trains, were regarded as very accessible (M = 4.15; SD = 0.95), the other recreation areas were assessed as more difficult to reach. The Altes Land which is accessible from the city centre by ferry was assessed significantly lower (M = 3.79; SD = 1.12). Interestingly the Lüneburg Heath, which is the recreation area furthest from Hamburg and only easily accessible by car, was assessed as more accessible (M = 3.70; SD = 1.04) than the Elbe Marshes (M = 3.61; SD = 1.08) which are directly adjacent to the south-eastern border of Hamburg, however not easily accessible by public transport.

Both service-related criteria were assessed significantly more positive for the Lüneburg Heath and the Altes Land, which are known all over Germany, than for the Harburg Hills and the Elbe Marshes, which are local or regional tourist destinations. Food services were assessed significantly different among the recreation areas (df = 3; F = 8.10; p<0.001). For the Lüneburg Heath (M = 3.66; SD = 0.97) and for the Altes Land (M = 3.47; SD = 0.99), it was assessed more positive than for the Harburg Hills (M = 2.84; SD = 1.05) and Elbe Marshes (M = 3.05; SD = 1.08). Information services were assessed significantly different among the recreation areas (df = 3; F = 330; p = 0.021). For the Lüneburg Heath (M = 3.44; SD = 0.96) and for the Altes Land (M = 3.14; SD = 1.14), information services were assessed more positive than for the Harburg Hills (M = 3.06; SD = 0.86) and Elbe Marshes (M = 2.89; SD = 1.09).

### Socio-demographic aspects of outdoor recreation

The only social factor which showed a fundamental influence on outdoor recreation was age. Other social factors like gender, educational level and place of residence were not significant. The importance of outdoor recreation within Hamburg was not evaluated significantly different among age groups (df = 3; F = 2.29; p = 0.078). In contrast, outdoor recreation outside of Hamburg was evaluated significantly different among age groups (df = 3; F = 4.22; p = 0.006). For older people outdoor recreation outside of Hamburg was much more important than for younger ones. The highest importance of recreation outside of Hamburg was found in the second oldest age group (50–65 years: M = 4.32). For this age group recreation outside of the city is exactly as important as recreation within the city. For the younger generations, recreation outside of the city was less important (<30 years: M = 3.82; 30–49 years: M = 3.99). For the oldest age group (≥66 years: M = 4.19), recreation outside of Hamburg was losing importance in comparison to the second oldest age group while the importance of recreation within Hamburg was still increasing.

The older the respondents were, the more likely it was that they knew the recreation areas and that they had visited the recreation areas. Also, the frequency of use increased significantly with age for all recreation areas (Lüneburg Heath df = 3; F = 4.05; p = 0.007; Harburg Hills df = 3; F = 13.75; p = <0.001; Elbe Marshes df = 3; F = 17.33; p<0.001; Altes Land df = 3; F = 13.33; p = <0.001).

All criteria were assessed significantly different among age groups except information services (diversity df = 3; F = 16.32; p<0.001; uniqueness df = 3; F = 11.96; p<0.001; naturalness df = 3; F = 3.39; p = 0.018; feeling of home df = 3; F = 3.98; p = 0.008; accessibility df = 3; F = 3.49; p = 0.016; food services df = 3; F = 7.86; p<0.001; information services df = 3; F = 2.020; p = 0.112). Older respondents generally assessed the criteria more positively than younger ones. There were only two exceptions; place attachment was assessed less positively in the age group 50–65 years than in the age group 30–49 years and accessibility was assessed less positively in the oldest age group ≥66 years than in the age group 50–65 years.

## Discussion

Overall, outdoor recreation was very important for urban dwellers. Especially outdoor recreation within the city was highly important and also carried out often. Although most people use parks and green spaces within the city more often than rural recreation areas outside of the city, the respondents stressed the importance of outdoor recreation outside of the city. This symbolic value of outdoor recreation outside of the city means that people cherish a landscape more because of its existence (existence value), than because of their frequent use of the landscape (value of use). Obviously, outdoor recreation sites outside of the city have a high symbolic value for urban dwellers besides of their value of use. The high symbolic value of recreation areas outside of the city might be due to the higher quality of the visit, namely higher aesthetic qualities of the rural areas or longer stays of the respondents in these areas. The high importance of outdoor recreation for urban dwellers found in this study is similar to other surveys which identified walking and hiking as the number one activity during day trips in the metropolitan region compared to other leisure activities [27]. For urban dwellers of Hamburg, walking and hiking is even more important than for the residents of the more rural counties in the metropolitan region [27].

A high symbolic value was found for specific recreation areas outside of Hamburg as there was no correlation between preference and use of recreation areas. Some of the recreation areas were highly valued, although they were not used more often than others, e.g. the Lüneburg Heath and the Altes Land. On the contrary, there were less preferred recreation areas, like the Harburg Hills and the Elbe Marshes, which were used as frequently as highly preferred recreation areas.

Concerning the characteristics of recreation areas, landscape-related criteria such as diversity, uniqueness, and naturalness (which indicate landscape attractiveness) were most important for urban dwellers. Especially, (perceived) naturalness was by far the most important criterion. Additionally, preferred recreation areas were characterised by positive ratings of uniqueness and naturalness. These results show that practical and service-related criteria, like the accessibility of recreation areas and the availability of service facilities are of minor importance. Although the results show the low importance of service-related infrastructure compared to landscape-related characteristics, it cannot be concluded that urban dwellers do not want service infrastructure at all. As we only asked for the most important criterion, it might be that urban dwellers regard service infrastructure as lower-ranking, but still as an important criterion. Vries & Boer [28] found in another survey on the local level in rural regions in the Netherlands (n = 702) that agricultural areas were more visited because of their proximity than because of their high quality, so that distance was an important factor while scenic beauty was not. The different results might be explained by the focus on different qualities and distances of recreation areas. While this study only considered recreational landscapes with a high aesthetic and recreational value, Vries & Boer [28] were looking at local farmland where the land use does not focus on recreational qualities at the highest priority. Results might be different for cities and regions, which do not have attractive landscapes in close proximity. Then, the factor naturalness might be less important, because the most important issue would be to have accessible recreation landscapes of any kind.

Furthermore, urban dwellers might have different preferences than people in rural areas. Hunziker [29] for example found that assessment results differ between experts, locals and tourists, especially for landscape change scenarios. Additionally, there might also be different preferences or even conflicts within the group of recreationists and tourists. This study focussed on quiet, nature based forms of outdoor recreation. In contrast, people who prefer other more infrastructure dependent outdoor activities like skiing or downhill mountain biking show different recreational preferences concerning infrastructure and landscape [30], [31]. Therefore, potentially different preferences of local inhabitants, farmers, tourists and other stakeholders who use different ecosystem services have to be considered when it comes to planning for recreation areas. Different planning approaches might also be necessary to consider the preferences of different age groups as age was the only social factor among gender, educational level and place of residence that significantly influenced outdoor recreation preferences and behaviour. While it might be more difficult to engage young people in outdoor recreation activities outside of the city, an option might be to focus on recreation activities within the city as young people have a higher preference to recreate there.

The study suggests that respondents' understanding of naturalness differs from an ecological definition. While urban dwellers perceived a similar degree of naturalness for all landscapes, the recreation areas have different degrees of human influence when taking account of naturalness and human influence as defined by Kowarik [32]. The orchards of the Altes Land are influenced by intensive agricultural use, while the heathland of the Lüneburg Heath, the meadows of the Elbe Marshes and the forests of the Harburg Hills are more natural. Therefore, perceived naturalness of whole landscapes does not seem to be directly dependent on the intensity of land use; it can rather be assumed that a landscape which is ‘green’ and without visual impairments of infrastructure and buildings is perceived as natural by most people. Boll et al. [33] found that agriculture and forestry are basically well accepted land uses in recreational areas; however, people clearly prefer less intensive agriculture, like grassland instead of fields. As all recreational landscapes that were considered in the study have a high aesthetic value in comparison to non-recreational landscapes, it is assumed that intensively-used agrarian areas would be evaluated less positively in terms of naturalness. Studies on a finer scale, which used photographs in the survey, show a more differentiated perception of naturalness. Lamb & Purcell [34] found in a survey (n = 81) that naturalness judgments were also dependent on vegetation structure. Their results showed that judgments of naturalness were related to ecological naturalness, but not equivalent.

The findings of this study suggest that the assessment of landscape aesthetics is not as subjective and individualistic as it is often claimed. Not only were landscape-related criteria evaluated more consistently among respondents, but also were more respondents able to assess landscape-related criteria than service and infrastructure-related criteria. These results are noteworthy as many authors regard landscape aesthetics as highly subjective [35], compared to measurable criteria like food and information services. Similar inter-subjective assessments might be due to the common cultural background of Hamburg residents. Hunziker [36] found that inter-subjective agreement among respondents increases, the larger and more complex the assessed landscape was. This study confirms the results of Hunziker as the assessed landscapes were whole recreation areas where the ‘mental’ picture of respondents was used instead of visualizations or photos.

As the size of the individual subsamples varies for some of the research questions, the results have to be interpreted with some care. While the whole survey included a robust sample size of 400 inhabitants of the Hamburg Metropolitan Region, the research questions on the four recreation areas were using subsamples for the individual recreation areas. The findings on the correlation between different criteria of the recreation areas were only based on four recreation areas in the southern Hamburg Metropolitan Region. Therefore, we regard the results that preferences for recreation areas are different from the actual use as a hypothesis, which has to be validated by further case studies.

The limitations of the methodology in this case study and the specific conditions of the survey have to be considered when generalizing the results. While Hamburg residents have many opportunities for their outdoor recreation within the city and several popular recreation areas in the immediate vicinity, the situation might be different in other cities. Therefore, it would be interesting to compare the results with other cities that have different endowment with green spaces within and outside of the city. The size of the city might also influence the relation of outdoor recreation within and outside of the city. While residents of large cities are expected to be more reliant on inner city recreation areas, residents of smaller cities might put an even stronger emphasis on recreation outside of the city. The number and quality of recreation areas might therefore influence the importance of outdoor recreation within and outside of the city.

## Conclusions

In metropolitan areas, it seems to be very important to provide outdoor recreation opportunities both within the city (e.g. parks) and in its proximity as in our study we found that for most people both alternatives are very important. Although urban dwellers recreate more often within the city, recreation outside of the city has a high symbolic value. Outdoor recreation outside of the city is even more important for older people, while younger people have a stronger focus to recreate inside the city.

As a higher preference for certain recreation areas did not automatically lead to higher frequency of use, there might be landscapes which are highly valued, but not used often. Thus, landscape changes in areas that are not used by many recreationists might as well provoke public protest. Hamburg residents use recreation areas like the Harburg Hills relatively frequently, although they were not the preferred landscape for the survey participants. On the contrary, recreation areas with a high preference like the Lüneburg Heath are used relatively infrequently by the majority of respondents.

For all recreational landscapes the actual appearance of the landscape is perceived as significantly more important for recreation than their accessibility and their endowment with service facilities. If a city has accessible and high value recreation areas in their surroundings, urban dwellers will appreciate this. Naturalness is by far the most important characteristic of recreation areas outside of the city. Concerning naturalness as perceived by people, it does not seem to be important to provide really natural areas without agricultural or silvicultural use, but areas that are green and not impaired by infrastructure and buildings.

## Supporting Information

Appendix S1 **Questionnaire.** A German version of the questionnaire was used in the survey in Hamburg (n = 400).
(PDF)
