# Supplementary material for: The Preference and Actual Use of Different Types of Rural Recreation Areas by Urban Dwellers—The Hamburg Case Study
Source: PLoS One. 2014 Oct 14;9(10):e108638. doi: 10.1371/journal.pone.0108638 (PMC4196755; doi:10.1371/journal.pone.0108638)
Supplement: Appendix S1 — Questionnaire. A German version of the questionnaire was used in the survey in Hamburg (n = 400). (PDF) [file pone.0108638.s001.pdf]

Questionnaire Hamburg

Interviewer:	Date:	Location:

Hello! My name is … and I am a Phd student/ student at the University of Hannover. We are conducting a research project on outdoor recreation. Would you be willing to take part in our survey for about 5-10 minutes?

May I ask you if you live in Hamburg or in the surrounding districts of Hamburg?

If no: As we are looking for respondents from Hamburg or the surrounding districts of Hamburg, we do not have further questions for you. Thank you very much for your willingness to take part in our survey! We wish you a nice day!

If yes: Just briefly for your information:
We define outdoor recreation as recreational activities that take place outdoors, like taking a walk, running, horseback riding, etc., where the attractiveness of nature and landscape plays an important role

And now the first question:
1)
a) Hamburg offers recreation areas inside the city like the Alster lake, the park Planten & Blomen and the Elbe river. Outside of Hamburg are for example the Lüneburg Heath, the Harburg Hills and the Elbe Marshes. Where does your outdoor recreation take part? *(show 1. answer card)*

almost exclusively within Hamburg	mainly inside Hamburg	roughly equal	mainly outside Hamburg	almost exclusively outside of Hamburg

b) How important is outdoor recreation within and outside of Hamburg for you personally? *(show 2. answer card)*

	5 very important	4 fairly important	3 moderately important	2 slightly important	1 not important
within Hamburg					
outside of Hamburg					

2) Now we are dealing with 4 recreation areas south of Hamburg: the Lüneburg Heath, the Harburg Hills, the Elbe Marshes and the Altes Land *(show 3. answer card)*
a) Do you know these recreation areas?
b) Have you visited these recreation areas?
If yes: c) How often do you use these recreation areas (e.g. for recreation, sports etc.)?
d) Which recreation area is, independently from your frequency of use, the most important for you?

	**a)**		**b)**		**c)**				**d)**
	yes	no	yes	no	never	weekly	monthly	yearly or less often	
Lüneburg Heath									
Harburg Hills									
Elbe Marshes									
Altes Land									

3) The [*Lüneburg Heath/…*] is most important for you.
a) How do you assess the following 7 aspects for [the *Lüneburger Heath/…*]? The assessment is not about right or wrong, but we are interested in your personal opinion. *(show 4. answer card)*
b) Which is for you, independently of [the *Lüneburg Heath/…*] the most important criterion of a recreation area?

	**a)**						**b)**
How do you assess…	5 very high	4 high	3 neither nor	2 low	1 very low	no idea	
… the diversity [of the Lüneburg Heath/…]?							
… the uniqueness?							
… the naturalness?							
… the place attachment?							
… the accessibility?							
… the food services?							
… the information services?							

---

4) We have some more questions for our statistical analysis:

a) What is the postcode of your place of residence? __________

b) How old are you? __________

c) Gender

male:	female:

d) What is your highest educational qualification?

University degree:	University entrance qualification:	middle school qualification:	basic school qualification:
